# Redetermination of pyridine-4-carbonitrile–chloranilic acid (1/1) at 180 K

**DOI:** 10.1107/S1600536808017182

**Published:** 2008-06-13

**Authors:** Kazuma Gotoh, Hirokazu Nagoshi, Hiroyuki Ishida

**Affiliations:** a Department of Chemistry, Faculty of Science, Okayama University, Okayama 700-8530, Japan

## Abstract

In the crystal structure of the title compound, C_6_H_4_N_2_·C_6_H_2_Cl_2_O_4_, two chloranilic acid (systematic name: 2,5-dichloro-3,6-dihydr­oxy-1,4-benzoquinone) mol­ecules are connected by O—H⋯O hydrogen bonds to form a dimeric unit. The pyridine-4-carbonitrile mol­ecules are linked on both sides of the dimer *via* N⋯H⋯O hydrogen bonds to give a centrosymmetric 2:2 complex of pyridine-4-carbonitrile and chloranilic acid. The H atom in the N⋯H⋯O hydrogen bond is disordered over two positions with approximately equal occupancies. The pyridine ring makes a dihedral angle of 61.54 (14)° with the chloranilic acid plane. The 2:2 units are further linked by inter­molecular C—H⋯O and C—H⋯Cl hydrogen bonds. This determination presents a siginficantly higher precision crystal structure than the previously published structure [Tomura & Yamasshita (2008 ▶). *X-ray Struct. Anal. Online*, **24**, x31–x32].

## Related literature

For related structures, see, for example: Gotoh, Asaji & Ishida (2007 ▶); Gotoh, Ishikawa & Ishida (2007 ▶); Tomura & Yamasshita (2008 ▶).
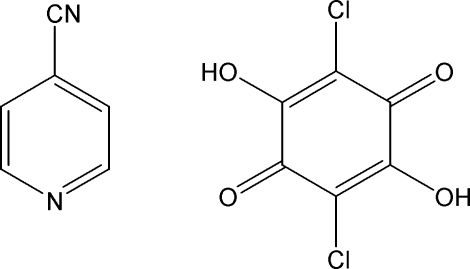


## Experimental

### 

#### Crystal data

C_6_H_4_N_2_·C_6_H_2_Cl_2_O_4_
*M* *_r_* = 313.10
Monoclinic, 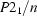

*a* = 14.9327 (8) Å
*b* = 4.9301 (3) Å
*c* = 17.0355 (10) Å
β = 93.0474 (18)°
*V* = 1252.37 (13) Å^3^
*Z* = 4
Mo *K*α radiation
μ = 0.53 mm^−1^
*T* = 180 (2) K
0.18 × 0.18 × 0.08 mm

#### Data collection

Rigaku R-AXIS RAPIDII diffractometer
Absorption correction: numerical (**ABSCOR**; Higashi, 1995 ▶) *T*_min_ = 0.900, *T*_max_ = 0.958
11539 measured reflections
3567 independent reflections
2165 reflections with *I* > 2σ(*I*)
*R*_int_ = 0.076

#### Refinement

*R*[*F* ^2^ > 2σ(*F* ^2^)] = 0.058
*wR*(*F* ^2^) = 0.176
*S* = 1.07
3567 reflections
192 parameters
H atoms treated by a mixture of independent and constrained refinement
Δρ_max_ = 0.43 e Å^−3^
Δρ_min_ = −0.73 e Å^−3^

### 

Data collection: *PROCESS-AUTO* (Rigaku/MSC, 2004 ▶); cell refinement: *PROCESS-AUTO*; data reduction: *CrystalStructure* (Rigaku/MSC, 2004 ▶); program(s) used to solve structure: *SHELXS97* (Sheldrick, 2008 ▶); program(s) used to refine structure: *SHELXL97* (Sheldrick, 2008 ▶); molecular graphics: *ORTEP-3* (Farrugia, 1997 ▶); software used to prepare material for publication: *CrystalStructure* and *PLATON* (Spek, 2003 ▶).

## Supplementary Material

Crystal structure: contains datablocks global, I. DOI: 10.1107/S1600536808017182/lh2639sup1.cif

Structure factors: contains datablocks I. DOI: 10.1107/S1600536808017182/lh2639Isup2.hkl

Additional supplementary materials:  crystallographic information; 3D view; checkCIF report

## Figures and Tables

**Table 1:** Hydrogen-bond geometry (Å, °)

*D*—H⋯*A*	*D*—H	H⋯*A*	*D*⋯*A*	*D*—H⋯*A*
O2—H2⋯N1	1.20 (10)	1.47 (10)	2.610 (3)	158 (7)
O4—H4⋯O1	0.78 (4)	2.21 (4)	2.661 (3)	118 (4)
O4—H4⋯O1^i^	0.78 (4)	1.99 (4)	2.656 (3)	144 (4)
N1—H1⋯O2	0.83 (12)	1.80 (13)	2.610 (3)	163 (10)
N1—H1⋯O3	0.83 (12)	2.45 (10)	2.957 (3)	120 (9)
C7—H7⋯Cl1^ii^	0.95	2.82	3.722 (3)	159
C8—H8⋯O4^iii^	0.95	2.46	3.324 (4)	151
C10—H10⋯Cl2^iv^	0.95	2.81	3.710 (3)	158
C11—H11⋯O3^v^	0.95	2.39	3.245 (4)	150

Symmetry codes: (i) 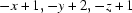; (ii) 
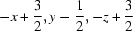
; (iii) 
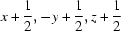
; (iv) 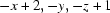; (v) 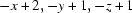.

**Fig. 1.**
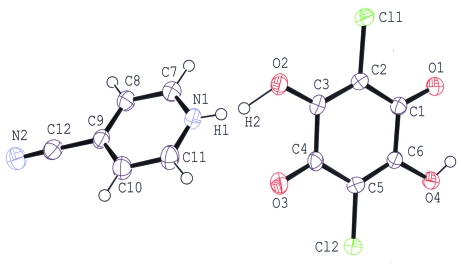
The molecular structure of (I), with the atom-labeling. Displacement ellipsoids of non-H atoms are drawn at the 50% probability level.

**Fig. 2.**
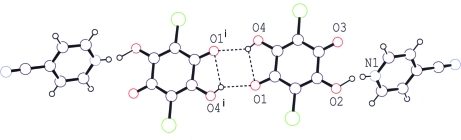
The structure of the 2:2 unit of (I). The dashed lines indicate O—H···O hydrogen bonds (symmetry codes as Table 1).

**Fig. 3.**
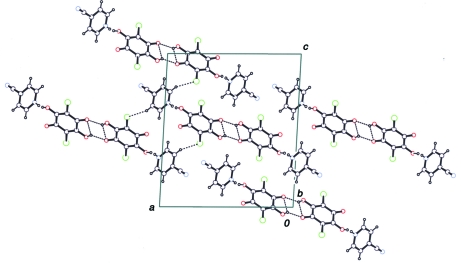
A packing diagram of (I), viewed down the b axis. The dotted lines indicate C—H···Cl hydrogen bonds.

## supplementary crystallographic information

### Comment

The title compound, (I), was prepared in order to extend our study on *D*—H···*A* hydrogen bonding (*D* = N, O, or C; *A* = N, O or Cl) in amine–chloranilic acid systems (Gotoh, Asaji & Ishida, 2007; Gotoh, Ishikawa & Ishida, 2007). This determination presents a siginficantly higher precision crystal structure than the previously published structure (Tomura & Yamasshita, 2008).

The asymmetric unit in (I) contains one pyridine-4-carbonitrile molecule and one chloranilic acid molecule (Fig. 1). Two chloranilic acid molecules related by an inversion centre are held together by O—H···O hydrogen bonds (Table 1) to form a dimer. The pyridine-4-carbonitrile molecules are linked on both sides of the dimer *via* N···H···O hydrogen bonds to give a centrosymmetric 2:2 complex of pyridine-4-carbonitrile and chloranilic acid (Fig. 2). The N···O distance is relatively short [2.610 (3) Å] and the H atom in the N···H···O hydrogen bond is disordered over two positions with site occupancies of 0.54 (17) and 0.46 (17). In the 2:2 unit, the pyridine and chloranilic acid planes are twised with a dihedral angle of 61.54 (14)°. The 2:2 units are further linked by C—H···O and C—H···Cl hydrogen bonds (Table 1 and Fig. 3).

### Experimental

Single crystals were obtained by slow evaporation from a methanol solution (30 ml) of chloranilic acid (500 mg) and pyridine-4-carbonitrile (250 mg).

### Refinement

C-bound H atoms were positioned geometrically (C—H = 0.95 Å) and refined as riding, with *U*_iso_(H) = 1.2*U*_eq_(C). The H atom in the O—H···O hydrogen bond was found in a difference Fourier map and refined isotropically (refined distances given in Table 1). The H atom in the N···H···O hydrogen bond was found to be disordered over two positions in a difference Fourier map. The positional parameters of the disordered H atom were refined, with *U*_iso_(H) = 1.2*U*_eq_(N, O) and the site occupancy factors were refined to 0.54 (17) and 0.46 (17).

### Crystal data

**Table:**

C_6_H_4_N_2_·C_6_H_2_Cl_2_O_4_	*F*_000_ = 632.00
*M**_r_* = 313.10	*D*_x_ = 1.660 Mg m^−^^3^
Monoclinic, *P*2_1_/*n*	Mo *K*α radiation λ = 0.71075 Å
Hall symbol: -P 2yn	Cell parameters from 7762 reflections
*a* = 14.9327 (8) Å	θ = 3.0–30.0º
*b* = 4.9301 (3) Å	µ = 0.53 mm^−^^1^
*c* = 17.0355 (10) Å	*T* = 180 (2) K
β = 93.0474 (18)º	Needle, brown
*V* = 1252.37 (13) Å^3^	0.18 × 0.18 × 0.08 mm
*Z* = 4	

### Data collection

**Table:**

Rigaku R-AXIS RAPIDII diffractometer	2165 reflections with *I* > 2σ(*I*)
Detector resolution: 10.00 pixels mm^-1^	*R*_int_ = 0.076
ω scans	θ_max_ = 30.0º
Absorption correction: numerical(*ABSCOR*; Higashi, 1995)	*h* = −20→20
*T*_min_ = 0.900, *T*_max_ = 0.958	*k* = −6→6
11539 measured reflections	*l* = −23→22
3567 independent reflections	

### Refinement

**Table:**

Refinement on *F*^2^	Hydrogen site location: inferred from neighbouring sites
*R*[*F*^2^ > 2σ(*F*^2^)] = 0.058	H atoms treated by a mixture of independent and constrained refinement
*wR*(*F*^2^) = 0.177	*w* = 1/[σ^2^(*F*_o_^2^) + (0.0805*P*)^2^] where *P* = (*F*_o_^2^ + 2*F*_c_^2^)/3
*S* = 1.07	(Δ/σ)_max_ = 0.001
3567 reflections	Δρ_max_ = 0.43 e Å^−^^3^
192 parameters	Δρ_min_ = −0.73 e Å^−^^3^
Primary atom site location: structure-invariant direct methods	Extinction correction: none
Secondary atom site location: difference Fourier map	

### Special details

**Table:**

Geometry. All e.s.d.'s (except the e.s.d. in the dihedral angle between two l.s. planes) are estimated using the full covariance matrix. The cell e.s.d.'s are taken into account individually in the estimation of e.s.d.'s in distances, angles and torsion angles; correlations between e.s.d.'s in cell parameters are only used when they are defined by crystal symmetry. An approximate (isotropic) treatment of cell e.s.d.'s is used for estimating e.s.d.'s involving l.s. planes.
Refinement. Refinement of *F*^2^ against ALL reflections. The weighted *R*-factor *wR* and goodness of fit *S* are based on *F*^2^, conventional *R*-factors *R* are based on *F*, with *F* set to zero for negative *F*^2^. The threshold expression of *F*^2^ > σ(*F*^2^) is used only for calculating *R*-factors(gt) *etc*. and is not relevant to the choice of reflections for refinement. *R*-factors based on *F*^2^ are statistically about twice as large as those based on *F*, and *R*- factors based on ALL data will be even larger.

### Fractional atomic coordinates and isotropic or equivalent isotropic displacement parameters (Å^2^)

**Table:**

	*x*	*y*	*z*	*U*_iso_*/*U*_eq_	Occ. (<1)
Cl1	0.72657 (5)	1.21818 (15)	0.67956 (4)	0.0293 (2)	
Cl2	0.72206 (5)	0.30951 (15)	0.41510 (4)	0.0317 (2)	
O1	0.57647 (13)	1.0461 (5)	0.56722 (13)	0.0341 (5)	
O2	0.87571 (13)	0.8329 (4)	0.64426 (13)	0.0303 (5)	
H2	0.914 (7)	0.62 (2)	0.647 (5)	0.036*	0.54 (17)
O3	0.87474 (13)	0.4764 (5)	0.52519 (13)	0.0343 (5)	
O4	0.57459 (14)	0.6729 (5)	0.45427 (14)	0.0316 (5)	
H4	0.540 (3)	0.781 (8)	0.466 (2)	0.048 (13)*	
N1	0.97525 (16)	0.4095 (6)	0.67823 (15)	0.0303 (6)	
H1	0.944 (9)	0.53 (3)	0.658 (6)	0.036*	0.46 (17)
N2	1.18662 (18)	−0.3815 (6)	0.78927 (17)	0.0370 (6)	
C1	0.64869 (18)	0.9233 (6)	0.56045 (16)	0.0256 (6)	
C2	0.72824 (18)	0.9664 (6)	0.60781 (16)	0.0249 (6)	
C3	0.80477 (18)	0.8142 (6)	0.60089 (17)	0.0248 (6)	
C4	0.80495 (18)	0.5996 (6)	0.53513 (17)	0.0254 (6)	
C5	0.72339 (19)	0.5549 (6)	0.48720 (16)	0.0266 (6)	
C6	0.65029 (18)	0.7065 (6)	0.49777 (17)	0.0244 (6)	
C7	0.9672 (2)	0.3253 (7)	0.7517 (2)	0.0354 (7)	
H7	0.9239	0.4070	0.7831	0.043*	
C8	1.0209 (2)	0.1210 (7)	0.78260 (18)	0.0333 (7)	
H8	1.0152	0.0599	0.8350	0.040*	
C9	1.08368 (18)	0.0063 (6)	0.73566 (17)	0.0275 (6)	
C10	1.0914 (2)	0.0969 (7)	0.65940 (19)	0.0372 (8)	
H10	1.1348	0.0208	0.6271	0.045*	
C11	1.0349 (2)	0.2994 (7)	0.6313 (2)	0.0355 (7)	
H11	1.0382	0.3613	0.5787	0.043*	
C12	1.1423 (2)	−0.2083 (6)	0.76598 (19)	0.0310 (7)	

### Atomic displacement parameters (Å^2^)

**Table:**

	*U*^11^	*U*^22^	*U*^33^	*U*^12^	*U*^13^	*U*^23^
Cl1	0.0338 (4)	0.0282 (4)	0.0255 (4)	−0.0001 (3)	−0.0033 (3)	−0.0035 (3)
Cl2	0.0369 (4)	0.0322 (4)	0.0257 (4)	0.0089 (3)	−0.0017 (3)	−0.0053 (3)
O1	0.0287 (10)	0.0399 (12)	0.0328 (12)	0.0106 (10)	−0.0062 (9)	−0.0093 (10)
O2	0.0283 (10)	0.0288 (11)	0.0327 (12)	0.0020 (9)	−0.0080 (9)	0.0018 (9)
O3	0.0287 (10)	0.0407 (13)	0.0333 (12)	0.0107 (9)	−0.0004 (9)	0.0012 (10)
O4	0.0242 (10)	0.0364 (12)	0.0335 (12)	0.0074 (9)	−0.0062 (9)	−0.0105 (10)
N1	0.0276 (13)	0.0333 (14)	0.0292 (14)	0.0032 (11)	−0.0055 (10)	0.0018 (12)
N2	0.0375 (14)	0.0337 (14)	0.0398 (16)	0.0036 (12)	0.0012 (12)	0.0039 (13)
C1	0.0263 (13)	0.0280 (14)	0.0223 (14)	0.0038 (12)	0.0001 (11)	0.0014 (12)
C2	0.0272 (13)	0.0264 (14)	0.0208 (13)	0.0010 (12)	−0.0011 (10)	−0.0012 (11)
C3	0.0212 (13)	0.0289 (15)	0.0239 (14)	−0.0010 (11)	−0.0030 (10)	0.0045 (12)
C4	0.0285 (14)	0.0241 (13)	0.0237 (14)	0.0044 (11)	0.0004 (11)	0.0062 (12)
C5	0.0305 (14)	0.0282 (14)	0.0209 (14)	0.0022 (12)	−0.0004 (11)	−0.0027 (12)
C6	0.0254 (14)	0.0239 (14)	0.0233 (14)	0.0020 (11)	−0.0039 (11)	−0.0003 (11)
C7	0.0345 (16)	0.0420 (19)	0.0294 (16)	0.0084 (14)	−0.0018 (13)	−0.0026 (14)
C8	0.0388 (16)	0.0361 (16)	0.0245 (15)	0.0075 (14)	−0.0023 (12)	0.0051 (14)
C9	0.0264 (13)	0.0268 (14)	0.0286 (15)	−0.0002 (12)	−0.0060 (11)	0.0001 (12)
C10	0.0332 (16)	0.0460 (19)	0.0323 (17)	0.0089 (15)	0.0016 (13)	0.0056 (15)
C11	0.0319 (16)	0.0425 (19)	0.0321 (17)	0.0059 (14)	0.0006 (13)	0.0106 (14)
C12	0.0327 (15)	0.0289 (15)	0.0309 (17)	−0.0018 (13)	−0.0030 (13)	0.0011 (13)

### Geometric parameters (Å, °)

**Table:**

Cl1—C2	1.743 (3)	C2—C3	1.377 (4)
Cl2—C5	1.723 (3)	C3—C4	1.541 (4)
O1—C1	1.247 (3)	C4—C5	1.447 (4)
O2—C3	1.262 (3)	C5—C6	1.343 (4)
O2—H2	1.20 (14)	C7—C8	1.375 (4)
O3—C4	1.226 (3)	C7—H7	0.9500
O4—C6	1.329 (3)	C8—C9	1.384 (4)
O4—H4	0.77 (4)	C8—H8	0.9500
N1—C7	1.330 (4)	C9—C10	1.384 (4)
N1—C11	1.343 (4)	C9—C12	1.451 (4)
N1—H1	0.83 (17)	C10—C11	1.376 (4)
N2—C12	1.138 (4)	C10—H10	0.9500
C1—C2	1.416 (4)	C11—H11	0.9500
C1—C6	1.512 (4)		
			
C3—O2—H2	110 (4)	C4—C5—Cl2	119.1 (2)
C6—O4—H4	110 (3)	O4—C6—C5	122.0 (3)
C7—N1—C11	122.0 (3)	O4—C6—C1	115.9 (2)
C7—N1—H2	119 (3)	C5—C6—C1	122.1 (2)
C11—N1—H2	119 (3)	N1—C7—C8	120.6 (3)
C7—N1—H1	123 (6)	N1—C7—H7	119.7
C11—N1—H1	115 (6)	C8—C7—H7	119.7
O1—C1—C2	125.3 (3)	C7—C8—C9	118.5 (3)
O1—C1—C6	117.0 (2)	C7—C8—H8	120.7
C2—C1—C6	117.7 (2)	C9—C8—H8	120.7
C3—C2—C1	123.0 (3)	C10—C9—C8	120.2 (3)
C3—C2—Cl1	119.4 (2)	C10—C9—C12	119.4 (3)
C1—C2—Cl1	117.6 (2)	C8—C9—C12	120.4 (3)
O2—C3—C2	125.8 (3)	C11—C10—C9	118.7 (3)
O2—C3—C4	116.2 (2)	C11—C10—H10	120.6
C2—C3—C4	118.0 (2)	C9—C10—H10	120.6
O3—C4—C5	122.9 (3)	N1—C11—C10	120.0 (3)
O3—C4—C3	118.4 (2)	N1—C11—H11	120.0
C5—C4—C3	118.7 (2)	C10—C11—H11	120.0
C6—C5—C4	120.4 (3)	N2—C12—C9	178.2 (3)
C6—C5—Cl2	120.5 (2)		
			
O1—C1—C2—C3	176.1 (3)	C4—C5—C6—O4	179.3 (3)
C6—C1—C2—C3	−2.4 (4)	Cl2—C5—C6—O4	0.6 (4)
O1—C1—C2—Cl1	−2.0 (4)	C4—C5—C6—C1	−1.5 (5)
C6—C1—C2—Cl1	179.5 (2)	Cl2—C5—C6—C1	179.9 (2)
C1—C2—C3—O2	−175.7 (3)	O1—C1—C6—O4	1.7 (4)
Cl1—C2—C3—O2	2.4 (4)	C2—C1—C6—O4	−179.7 (3)
C1—C2—C3—C4	3.8 (4)	O1—C1—C6—C5	−177.5 (3)
Cl1—C2—C3—C4	−178.1 (2)	C2—C1—C6—C5	1.1 (4)
O2—C3—C4—O3	−5.3 (4)	C11—N1—C7—C8	0.5 (5)
C2—C3—C4—O3	175.1 (3)	N1—C7—C8—C9	0.3 (5)
O2—C3—C4—C5	175.5 (3)	C7—C8—C9—C10	−0.1 (5)
C2—C3—C4—C5	−4.1 (4)	C7—C8—C9—C12	179.5 (3)
O3—C4—C5—C6	−176.2 (3)	C8—C9—C10—C11	−0.8 (5)
C3—C4—C5—C6	3.0 (4)	C12—C9—C10—C11	179.6 (3)
O3—C4—C5—Cl2	2.5 (4)	C7—N1—C11—C10	−1.4 (5)
C3—C4—C5—Cl2	−178.4 (2)	C9—C10—C11—N1	1.5 (5)

### Hydrogen-bond geometry (Å, °)

**Table:**

*D*—H···*A*	*D*—H	H···*A*	*D*···*A*	*D*—H···*A*
O2—H2···N1	1.20 (10)	1.47 (10)	2.610 (3)	158 (7)
O4—H4···O1	0.78 (4)	2.21 (4)	2.661 (3)	118 (4)
O4—H4···O1^i^	0.78 (4)	1.99 (4)	2.656 (3)	144 (4)
N1—H1···O2	0.83 (12)	1.80 (13)	2.610 (3)	163 (10)
N1—H1···O3	0.83 (12)	2.45 (10)	2.957 (3)	120 (9)
C7—H7···Cl1^ii^	0.95	2.82	3.722 (3)	159
C8—H8···O4^iii^	0.95	2.46	3.324 (4)	151
C10—H10···Cl2^iv^	0.95	2.81	3.710 (3)	158
C11—H11···O3^v^	0.95	2.39	3.245 (4)	150

Symmetry codes: (i) −*x*+1, −*y*+2, −*z*+1; (ii) −*x*+3/2, *y*−1/2, −*z*+3/2; (iii) *x*+1/2, −*y*+1/2, *z*+1/2; (iv) −*x*+2, −*y*, −*z*+1; (v) −*x*+2, −*y*+1, −*z*+1.
